# MicroRNA-572 expression in multiple sclerosis patients with different patterns of clinical progression

**DOI:** 10.1186/s12967-015-0504-2

**Published:** 2015-05-07

**Authors:** Roberta Mancuso, Ambra Hernis, Simone Agostini, Marco Rovaris, Domenico Caputo, Mario Clerici

**Affiliations:** Don C. Gnocchi Foundation – ONLUS, P.zza Morandi, 3, 20100 Milano, Italy; Department of Physiopathology and Transplantation, University of Milano, Milano, Italy

**Keywords:** microRNA, Multiple Sclerosis, NCAM, Remyelination, Disability, Serum

## Abstract

**Background:**

Demyelination and failure of remyelination are core mechanisms in the pathogenesis of multiple sclerosis (MS); the factor(s) modulating these processes are still mostly unknown. MicroRNA 572 (miR-572) is deregulated in MS and is suggested to targets neural cell adhesion molecule (NCAM), a glycoprotein involved in CNS reparative mechanisms. The aim of this study is to analyze miR-572 in patients with different clinical phenotypes of MS.

**Methods:**

qPCR quantification of miR-572 isolated from serum was performed in 16 primary progressive (PP), 15 secondary progressive (SP), 31 relapsing remitting (RR) MS patients and 15 sex-and age-matched healthy controls.

**Results:**

miR-572 expression was reduced overall in MS patients (p < 0.05) compared to HC; this miRNA was significantly upregulated in SPMS and in RRMS during disease relapse, whereas it was downregulated in PPMS and in quiescent phases of RRMS. miR-572 expression correlated with EDSS scores (R_Sp_ = 0.491; p < 0.05) independently of the clinical phenotype. The results suggest that this miRNA might be a tool that helps distinguishing between PPMS and SPMS and between relapsing and remitting phases in RRMS.

**Conclusions:**

Evaluation of miR-572 may serve as a non-invasive biomarker for remyelination.

**Electronic supplementary material:**

The online version of this article (doi:10.1186/s12967-015-0504-2) contains supplementary material, which is available to authorized users.

## Background

Multiple Sclerosis (MS) is an inflammatory neurodegenerative disorder of the central nervous system (CNS) of unknown etiology characterized by a high degree of heterogeneity with respect to clinical manifestations and response to treatment. The pathological process common to all forms of this disease is the inadequate repair of myelin damage in CNS, a complex and yet mostly unclear process that becomes increasingly evident as MS progresses [1]. Many reasons, including the inadequate recruitment of oligodendrocytes precursor cells (OPC) and an impairment in the processes leading OPC to differentiate into oligodendrocytes, can hamper the remyelination process. The accumulation of myelin alterations leads to axonal degeneration; this appears early in disease, but becomes prominent in the progressive form of MS [2,3], leading to further axonal and neuronal loss and progression of disability.

Transient remyelination [4] and at least a partial restoration of conduction velocity [5] is seen in MS, processes that can protect axons from injury and determine the temporary remission of disease in RRMS [6]**.** The efficacy of the remyelination process varies widely in different patients and in different phases of the disease. Thus, although remyelination is frequently more efficient in the early stage of MS [4], extensive remyelination has been sometimes observed in chronic patients [7], even if this phenomenon is usually rare or absent in late chronic disease [8]. The degree of remyelination also differs in the diverse clinical phenotypes of MS. Thus, a more complete remyelination is known to occur in the brain of primary progressive (PPMS) compared to secondary progressive (SPMS) patients [9], a finding that might explain the relative preservation of cognitive function in PPMS patients [10].

Although the molecular mechanisms responsible for remyelination are still mostly unclear, the neural cell adhesion molecule (NCAM or CD56) is suspected to play an important role in this process, as this protein, that belongs to the immunoglobulin super-family, is linked to CNS reparative mechanism [11]. NCAM is an integral membrane glycoprotein in neuronal and glial cells and it promotes adhesion between cells through interacting with homologous molecules on adjacent cells. Notably, binding of NCAM to polysialic acid (PSA-NCAM) decreases cell adhesion, inducing structural remodeling in brain during the development of the nervous system [12] and contributing to the formation of axonal networks [13]. In the adult nervous system NCAM is expressed in limited brain areas, where it is involved in neuronal sprouting and synaptic remodeling [14], while PSA-NCAM acts as a negative signal of myelination [15]. Defective myelin compaction was observed in NCAM deficient mice [16], and deletion of chromosome coding sequence for NCAM in children has been associated with delayed myelination [17]. Finally, NCAM is differentially expressed in active or chronic MS lesions [18], and abnormal levels of soluble NCAM (NCAMs) were suggested to be associated with the progression of disability in MS [19,20].

Data stemming from a computational approach (TargetScan) showed that NCAM is a possible target for microRNA (miRNA) 572, a miRNA that was recently observed to be deregulated in MS [21]. As is the case with miRNAs, the result of the miR-572/NCAM interaction would be the modulation of NCAM activity, and this would correlate with the degree of remyelination. We evaluated the levels of this miRNA in serum of a group of MS patients with different clinical forms, analyzing possible correlations with progression of disease and clinical characteristics.

## Methods

### Patients and samples collection

Thirty-one chronic progressive (16 primary progressive –PPMS– and 15 secondary progressive –SPMS) and 31 relapsing remitting (RRMS) patients as well as 15 age-and sex-matched healthy controls (HC) were enrolled for the study after signing an informed consent approved by the Ethics Committee of the Don C. Gnocchi Foundation-ONLUS in Milano, Italy. Among the RRMS patients, 15 were undergoing clinical relapse of the disease whereas the other 16 were in a clinically stable phase without areas of enhancement at the time of enrolment, as demonstrated by magnetic resonance imaging (MRI) with gadolinium.

All the patients were diagnosed according to the revised McDonald criteria [22] and did not receive any treatment for at least two months before the blood withdrawal. RRMS patients in whom clinical relapse was observed underwent blood collection before the first glucocorticoid infusion. Demographic and clinical characteristics of all the subjects enrolled are shown in Table 1. Serum samples were obtained from whole blood at the end of clotting time (60 minutes) by centrifugation (3400 *g* × 10 minutes).

**Table 1:** Demographic and clinical characteristics of the individuals enrolled in the study

	**PPMS**	**SPMS**	**Stable RRMS**	**Acute RRMS**	**Controls**	**p value**
N	16	15	16	15	15	
Gender (M:F)	9:7	7:8	3:13	7:8	2:13	ns*
Age, yrs	52.8 ± 10.3	50.8 ± 8.0	42.8 ± 10.3	38.7 ± 8.4	38.7 ± 9.8	p < 0.01^#^
Disease duration, yrs	13.4 ± 8.2	10.6 ± 9.9	10.6 ± 9.9	11.3 ± 8.0	/	*ns* ^*#*^
EDSS	6.36 ± 1.63	6.64 ± 1.40	2.25 ± 2.11	3.73 ± 1.90	/	p < 0.01^*#*^

Mean value ± standard deviation are showed; MS: multiple sclerosis; PP: primary progressive; SP: secondary progressive; RR: relapsing remitting; EDSS: Kurtzke Expanded Disability Status Scale; *Fisher’s exact test; ^*#*^Anova test.

### miRNA target prediction

TargetScan (http://www.targetscan.org/), and MiRanda (http://www.microrna.org/microrna/home.do) bioinformatic tools were utilized to determine miRNA potential target mRNAs.

### miRNA isolation and cDNA retrotranscription

miRNA isolation from serum was performed with a column based kit (miRNeasy Mini kit, Qiagen GmbH, Hilden, Germany) according to the manufacturer’s specific protocol. Notably, as the yield of RNA from small volume serum samples was below the limit of quantitation by spectrophotometry, prior to miRNA extraction, 1 μg of carrier RNA (MS2 RNA, Roche Life Science, Mannheim, Germany) and 5 μl of non-human (*C. elegans*) synthetic miR-39 (*C.el.*-miR-39) (5 nM) were added to 200 μl of serum after denaturation with Qiazol Lysis Reagent. Total RNA was eluted in 30 μl.

The inclusion of the synthetic miRNA was necessary for adjusting for differences in efficiency of RNA recovery between samples. Four μl of RNA was utilized for retro-transcription reactions (in a final volume of 20 μl), performed in triplicate using the universal cDNA synthesis kit (miRCURY LNA™ Universal cDNA synthesis kit, Exiqon Inc., Vedbaek, Denmark). Efficiency of cDNA synthesis and absence of qPCR inhibitors were monitored for all the samples by addition of the synthetic control template (RNA spike-in, Exiqon Inc.). To avoid variation due to sample differences and handling, all the variables involved in the procedure were kept consistent throughout the study.

### miRNA selection and quantitative PCR assay

The study focused on a specific miRNA (miR-572) previously identified as being upregulated in plasma of MS patients [21]. A specific LNA™-individual microRNAs assay (Exiqon Inc.) was utilized to detect in sera the miRNA target (hsa-miR-572; cat. 204696), reference miRNAs, and the haemolysis control (hsa-miR-16; cat. 204409) according to the manufacturer’s instructions. A set of 3 endogenous miRNAs was selected as candidate reference genes to normalize the data: miR-103 (Exiqon, cat. 204063), -191 (Exiqon, cat. 204306), −423 (Exiqon, cat. 204593). A synthetic *C.el.* miR-39 (Exiqon, cat. 203952) was also used to normalize the results.

Briefly, qPCR amplification was performed on real time PCR system (Step One, Applied Biosystem, Foster City, CA) in 10 μl of reaction mix containing SYBR GREEN master mix (Exiqon Inc.), specific primer set for each miRNA and 4 μl of cDNA. Each cDNA template was tested in triplicate by qPCR. Negative controls, without rt-template controls, and no-template controls were included in each session. An additional step in the qPCR analysis was performed to evaluate the specificity of the amplification products by generating a melting curve for each reaction.

### Data processing and statistical analysis

Manual baseline and threshold were set manually on the instrument for the evaluation of row Cq value for each sample. Because of the scarcity of miRNA in serum, Cq = 38 was set as the cut-off.

The NormFinder algorithm was used to calculate the expression stabilities of the candidate reference genes. NormFinder calculates the stabilities of candidate reference genes based on the intra- and inter-group variations. A lower stability value indicates a more stably expressed gene [23].

Relative quantification was determined by the comparative delta-Cq method using the more stable reference miRNA (ref) indicated by NormFinder for median normalization procedure: (Raw Cq value - [(ref miRNA average Cq of the given sample) - (ref miRNA median Cq value)]; fold expression levels (2^-ΔΔCq^) were calculated as described [24]; fold change < 0.5 was indicative of down-regulation and > 2 of up-regulation. Absence of qPCR inhibition for haemolysis was verified monitoring the stability of Cq of miR-16, commonly found in red blood cells.

Statistical analyses were accomplished using commercial software (MedCalc®, version 11.5.0.0).

Demographic and clinical quantitative data, reported as mean and standard deviation, were analyzed by one-way analysis of variance (ANOVA). The others quantitative variable, not normally distributed, are expressed as median and 95% confidence interval (CI). Logarithmic transformation was applied to miR-572 relative expression fold, and Kruskal-Wallis was used to compare value among groups, whereas Mann Whitney test was used to determine the significance between two groups. Spearman’s rank correlation coefficient was used in the correlation analysis between miR-572 and clinical variables. p values < 0.05 were considered statistically significant. Receiver operating characteristics analysis (ROC) and area under curve (AUC) were used to evaluate the potential of miRNA as biomarker (see Additional file 1).

## Results

### Selection of candidate reference genes

*C.el* miR-39 was selected for normalization because NormFinder ranked it as the most stably expressed gene, followed by miR-103, miR-423, and miR-191.

Moreover, normalization with two other miRNAs (miR-103, miR-423) was performed to confirm the analysis obtained with *C.el* miR-39.

### miR-572 expression levels in MS patients and in HC

The expression levels of circulating miR-572 were evaluated in MS patients with different clinical disease phenotypes as well as in HC. Demographic and clinical details showed that disease duration was comparable for all groups, although patients’ age was significantly higher in progressive than in RRMS (Table 1).

**Figure 1:**
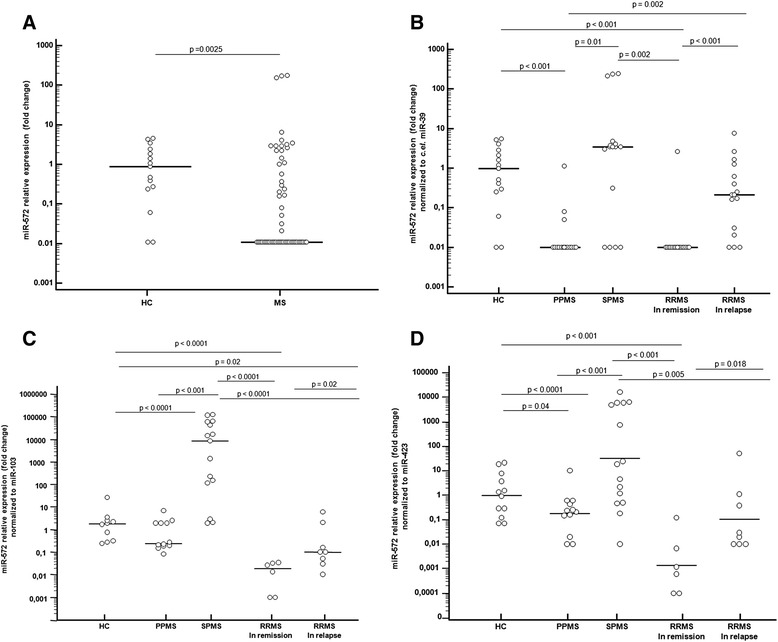
miR-572 expression level in serum of MS patients and controls. miR-572 relative expression fold change (ref: *C. el.* miR-39) in serum of MS patients and healthy controls (HC) **(panel A)** and of MS patients with different disease phenotypes, using as reference *C. el* miR-39 **(panel B)**, miR-103 **(panel C)** and miR-423 **(panel D)**. Analyses reported in **panel C** and **D** were performed on a subgroup of subjects. PPMS = primary progressive MS; SPMS = secondary progressive MS; RRMS = relapsing-remitting MS. Bars represent the median values; statistical significance is shown.

Results showed that the serum concentration of miR-572 was significantly down regulated in the overall group of MS patients (median fold: 0.01; 95% CI: 0.01-0.05) compared to HC (0.98; 0.26-2.63, p = 0.0025) (Figure 1, Panel A).

### miR-572 expression levels in MS patients with different disease phenotype

Notably, a wide variation of miR-572 expression levels was observed when miR-572 serum concentration was compared in MS patients with different patterns of disease. Thus, the lowest serum concentration of this miRNA was detected in PPMS (0.01; 0.01-0.012) and in RRMS patients during the remitting phase of disease (0.01; 0.01-0.01); the values observed in these cases were significantly lower than those seen in HC (p < 0.001 in both cases). Conversely, serum concentration of miR-572 was significantly increased in SPMS (3.4; 0.01-212.94) compared to PPMS patients (p = 0.01), and in RRMS patients undergoing a disease relapse (0.21; 0.02-1.03) in comparison to those RRMS patients evaluated during remission (p < 0.001) (Figure 1, Panel B). In Figure 1, panels C represents miR-572 expression after normalization with miR-103; results in panel D are normalized with miR-423.

### Correlations between miR-572 serum concentrations and clinical parameters

**Figure 2:**
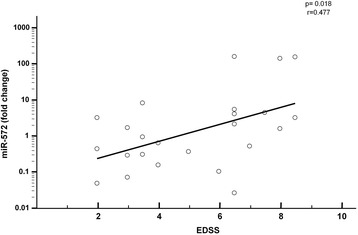
Correlation between miR-572 and disability in MS patients. Correlation between miR-572 relative expression fold change in serum and Kurtzke Expanded Disability Status Scale (EDSS) in MS patients.

The Kurtzke Expanded Disability Status Scale (EDSS) is a widely accepted method of quantifying disability in MS; possible correlations between EDSS scores and miR-572 serum concentrations were analyzed. Considering the overall MS population, a significant positive correlation independently from the clinical phenotype was observed between relative expression of miR-572 in serum and EDSS disability score (R_s_ = 0.477; p = 0.018) (Figure 2); in contrast with these results, disease duration was not associated with miR-572 serum concentration.

## Discussion

Circulating miRNAs have emerged as potential biomarkers for several human diseases including MS [25,26]. In the present study the expression levels of miR-572 were evaluated in serum of MS patients with different patterns of disease. We focused on this molecule because of data indicating that circulating levels of miR-572 are increased in MS patients [21] and because a putative target for miR-572 is the neuronal cell adhesion molecule (NCAM), a protein involved in the maturation of the nervous system [27], and more recently also suggested to play a role in neurodegenerative diseases. In fact many evidences showed the influence of NCAM in neurite outgrowth [28], synaptic plasticity [29] and CNS repair and remyelination [30].

Results showed that miR-572 was down-regulated in the MS patients compared to HC subjects, a phenomenon already evidenced in previous analyses on other miRNAs [26,31]; interestingly, a modulation of miR-572 was observed upon clustering of MS patients in accordance with clinical course. Thus, comparisons between groups showed that serum levels of miR-572 were markedly different when the primary and secondary forms of progressive MS were compared, with significant increase in SPMS and significant decrease in PPMS. Similarly, in RRMS patients a significant increase of serum concentrations of miR-572 was observed in relapsing compared to remitting patients.

It is known that a major challenge for the analysis of circulatory miRNA is data normalization, a crucial point for an objective evaluation of their expression level and to avoid the introduction of systematic bias in the analysis. The strategy we adopted was to use a synthetic spike-in oligonucleotide, as reported by several other papers [32,33]. Thus, *C. el* miR-39 was selected as reference because indicated as the best candidate by Normfinder software. To further confirm our results two other miRNA (miR-103, miR-423) were used singularly to normalize the data; the three methods led to the same results.

Plasma levels of miR-572 were previously reported to be increased in MS patients [21]. The discrepancy between these and our results can be explained considering that the data reported by Siegel et al. stem from analyses performed in a very small group (n = 4) of MS patients that were not clinically classified. Moreover, significant variation in specificity among different qPCR platform/methods is a well known problem as less abundant miRNAs can escape detection with technologies such as microarray, cloning and hybridization [34]. Thus, the choice of the method used (qPCR array *vs.* individual qPCR with LNA™-primers), or of different biological samples (serum vs. plasma) as well as the clinical course of analyzed patients can explain these discrepancies.

Different studies have investigated whether serum concentration of soluble NCAM (NCAMs) could be used as a biomarker in human pathology [35]. Particularly, in MS the low levels of NCAMs detected in the cerebrospinal fluid (CSF) suggest that this molecule could contribute to the decreased CNS repair observed in this disease [36,37]. Notably, recent data showed the presence of a negative correlation between EDSS scores and CSF NCAMs levels, and demonstrated that the concentration of NCAMs decreases with disease progression [19]. On the contrary, increasing CSF NCAMs levels are observed in RRMS patients after treatment of the acute phase with corticosteroids [36], paralleling the progressive clinical improvement seen after the cessation of relapses, and indicating a possible relation with myelin repair mechanism.

Understanding the mechanism(s) responsible for the modulation of NCAM thus could shed light on the pathogenesis of MS and, possibly, open novel therapeutic avenues for this disease. In the attempt to clarify this issue we analyzed serum levels of miR-572, a miRNA that is suggested by computational approaches to bind NCAM and, thus, regulates its activity. Results herein are the first in which circulating levels of miR-572 are analyzed in a large group of MS patients subdivided according to the disease course. Because an increase of miRNA is mostly associated with expression and activity reduction of the target protein [38], the correlation between serum levels of miR-572 and MS clinical phenotypes allows the speculation that the observed changes in miR-572 may result in different degrees of myelin repair activity. This hypothesis is supported by the fact that the concentration of miR-572 was significantly reduced (hence NCAM activity was likely significantly increased) in PPMS patients, in whom a more complete remyelination is known to occur [9]. Further support to this possibility stems from the observation that initiation of steroids in RRMS patients with disease relapses resulted in the reduction of serum concentration of miR-572 (our data) and a parallel increase of NCAMs in CSF [36]. Finally, a positive correlation was observed between serum miR-572 levels and disability score in MS patients; these results are specular to the recently reported inverse correlation detected in MS between NCAMs in CSF and EDSS scores [19]. An important weakness of the study is the limited number of analyzed patients; this could have influenced the results. Further analyses with a larger cohort will be necessary to confirm these preliminary data and to validate experimentally the miR-572/NCAM target interaction and modulation.

## Conclusions

Our results suggest that miR-572 could be one of the regulatory factors involved in the remyelination process and raise the possibility that the changes in miR-572 expression seen in MS patients with different patterns of disease may play a role in such process and could be used as a biomarker.

## Additional file

Additional file 1:
**Figure S1. Receiver operating characteristic analysis of miR-572 for discriminating between MS and HC.** To evaluate the potential of miRNA as biomarkers, receiver operating characteristics analysis (ROC) and area under curve (AUC) were performed. Results showed that serum levels of miR-572 have a predictive value in distinguishing MS from HC (AUC: 0.741; 95% CI: 0.623-0.834; p= 0.0003) and PPMS from SPMS (AUC: 0.765; 95% CI: 0.544-0.915; p=0.0067)**.** Moreover serum miR-572 concentrations could clearly distinguish between acute and stable disease in RRMS patients (AUC 0.848; 95% CI: 0.674-0.951; p<0.0001). ROC curves of serum concentration of miR-572 in MS patients and HC (panel A), in PPMS compared to SPMS patients (panel B) and in relapsing compared to remitting MS (panel C). AUC: area under curve; CI: confidence interval. For Methods see main text.

## Abbreviations

ANOVA: One-way analysis of variance
AUC: Area under curve
cDNA: Complementary DNA
CI: Confidence interval
CNS: Central nervous system
Cq: Quantification cycle
CSF: Cerebrospinal fluid
EDSS: Kurtzke Expanded Disability Status Scale
HC: Healthy control
LNA^TM^: Locked nucleic acid
miRNA: microRNAs
MRI: Magnetic resonance imaging
MS: Multiple Sclerosis
NCAM: Neural cell adhesion molecule
NCAMs: Soluble NCAM
OPC: Oligodendrocytes precursor cells
PPMS: Primary progressive MS
PSA-NCAM: Polysialylated NCAM
qPCR: Quantitative polymerase chain reaction
ROC: Receiver operating characteristics analysis
RRMS: Relapsing remitting MS
SPMS: Secondary progressive MS
