# Supplementary material for: MicroRNA-572 expression in multiple sclerosis patients with different patterns of clinical progression
Source: J Transl Med. 2015 May 7;13:148. doi: 10.1186/s12967-015-0504-2 (PMC4429409; doi:10.1186/s12967-015-0504-2)
Supplement: Additional file 1: — Figure S1. Receiver operating characteristic analysis of miR-572 for discriminating between MS and HC. To evaluate the potential of miRNA as biomarkers, receiver operating characteristics analysis (ROC) and area under curve (AUC) were performed. Results showed that serum levels of miR-572 have a predictive value in distinguishing MS from HC (AUC: 0.741; 95% CI: 0.623-0.834; p= 0.0003) and PPMS from SPMS (AUC: 0.765; 95% CI: 0.544-0.915; p=0.0067). Moreover serum miR-572 concentrations could clearly distinguish between acute and stable disease in RRMS patients (AUC 0.848; 95% CI: 0.674-0.951; p<0.0001). ROC curves of serum concentration of miR-572 in MS patients and HC (panel A), in PPMS compared to SPMS patients (panel B) and in relapsing compared to remitting MS (panel C). AUC: area under curve; CI: confidence interval. For Methods see main text. [file 12967_2015_504_MOESM1_ESM.docx]

**MicroRNA-572 Expression in Multiple Sclerosis Patients**

**with Different Patterns of Clinical Progression**

Mancuso R^1^, Hernis A^1^, Agostini S^1^, Rovaris M^1^, Caputo D^1^, Clerici M^1,2^.

^1^Don C. Gnocchi Foundation – ONLUS, Milano, Italy

^2^Department of Physiopathology and Transplantation, University of Milano, Milano, Italy.

**Additional file**

**Figure 1S: Receiver operating characteristic analysis of miR-572 for discriminating between MS and HC.** To evaluate the potential of miRNA as biomarkers, receiver operating characteristics analysis (ROC) and area under curve (AUC) were performed. Results showed that serum levels of miR-572 have a predictive value in distinguishing MS from HC (AUC: 0.741; 95% CI: 0.623-0.834; p= 0.0003) and PPMS from SPMS (AUC: 0.765; 95% CI: 0.544-0.915; p=0.0067)**.** Moreover serum miR-572 concentrations could clearly distinguish between acute and stable disease in RRMS patients (AUC 0.848; 95% CI: 0.674-0.951; p<0.0001). ROC curves of serum concentration of miR-572 in MS patients and HC (panel A), in PPMS compared to SPMS patients (panel B) and in relapsing compared to remitting MS (panel C). AUC: area under curve; CI: confidence interval. For Methods see main text.
